# The Promoting Effect of Gut Microbiota on Growth and Development of Red Palm Weevil, *Rhynchophorus ferrugineus* (Olivier) (Coleoptera: Dryophthoridae) by Modulating Its Nutritional Metabolism

**DOI:** 10.3389/fmicb.2019.01212

**Published:** 2019-05-29

**Authors:** Prosper Habineza, Abrar Muhammad, Tianliang Ji, Rong Xiao, Xianyuan Yin, Youming Hou, Zhanghong Shi

**Affiliations:** ^1^State Key Laboratory of Ecological Pest Control for Fujian and Taiwan Crops, Fujian Agriculture and Forestry University, Fujian, China; ^2^Fujian Provincial Key Laboratory of Insect Ecology, College of Plant Protection, Fujian Agriculture and Forestry University, Fujian, China

**Keywords:** *Rhynchophorus ferrugineus*, gut microbiota, insect symbiosis, symbiotic invasion, nutrition metabolism, germ-free larvae

## Abstract

Red palm weevil (RPW), *Rhynchophorus ferrugineus* Olivier, is a destructive pest for palm trees worldwide. Recent studies have shown that RPW gut is colonized by microbes and alterations in gut microbiota can significantly modify its hemolymph nutrition content. However, the exact effects of gut microbiota on RPW phenotype and the underlying mechanisms remain elusive. Here germ-free (GF) RPW larvae were generated from dechorionated eggs which were reared on sterilized artificial food under axenic conditions. Compared with controls, the larval development of GF RPW individuals was markedly depressed and their body mass was reduced as well. Furthermore, the content of hemolymph protein, glucose and triglyceride were dropped significantly in GF RPW larvae. Interestingly, introducing gut microbiota into GF individuals could significantly increase the levels of the three nutrition indices. Additionally, it has also been demonstrated that RPW larvae monoassociated with *Lactococcus lactis* exhibited the same level of protein content with the CR (conventionally reared) insects while feeding *Enterobacter cloacae* to GF larvae increased their hemolymph triglyceride and glucose content markedly. Consequently, our findings suggest that gut microbiota profoundly affect the development of this pest by regulating its nutrition metabolism and different gut bacterial species show distinct impact on host physiology. Taken together, the establishment of GF and gnotobiotic RPW larvae will advance the elucidation of molecular mechanisms behind the interactions between RPW and its gut microbiota.

## Introduction

It is ubiquitous that animal hosts, including insects, have established symbiotic interactions with microbes. Increasing evidence from invertebrate model organism *Drosophila melanogaster* have indicated that intestinal microbes can impact many host physiological traits such as development, immunity, maturation, longevity and nutrition (Shin et al., 2011; Storelli et al., 2011; Wong et al., 2014; Strigini and Leulier, 2016) and fitness-related behaviors including mating preference (Sharon et al., 2011), foraging decision (Wong et al., 2017) and social communications (Venu et al., 2014). Interestingly, the similar profound effects of commensal microbiota on growth, health and disease of honey bee, *Apis mellifera*, have been also determined recently (Zheng et al., 2017; Raymann and Moran, 2018). In recent years, the diversity and composition of gut microbiota associated with some notorious pests, containing the cotton bollworm *Helicoverpa armigera* (Xiang et al., 2006), Oriental fruit fly *Bactrocera dorsalis* Hendel (Shi et al., 2012), Diamondback moth *Plutella xylostella* L. (Xia et al., 2013), Red turpentine beetle *Dendroctonus valens* LeConte (Zhou F. et al., 2017; Cheng et al., 2018) and *Spodoptera littoralis* (Chen et al., 2016), have been intensively investigated.

Unfortunately, the physiological mechanisms by which these insect pests maintain the symbiotic interactions with their gut commensal bacteria are still poorly understood. It is increasingly recognized that one means by which intestinal microbiota communicate with their hosts is through metabolic byproducts that arise from bacterial catabolism of host diet (Kamareddine et al., 2018). Gut microbiota derived short chain fatty acids (SCFAs) have been confirmed to be crucial for host health by executing important metabolic functions (Canfora et al., 2015; Koh et al., 2016; Fellows et al., 2018). For instance, these metabolites are recognized by specific G protein-coupled receptors on enteroendocrine cells, and then these cells release small enteroendocrine peptides to modulate local and systemic lipid and carbohydrate metabolism to maintain host homeostasis (Bolognini et al., 2016; Miyamoto et al., 2016). Recently, a study on *D*. *melanogaster* has revealed that a SCFA, acetate, increases the secretion of the endocrine peptide Tachykinin to maintain the timely larval development and optimal lipid metabolism (Kamareddine et al., 2018). Insecta is the most diverse biological group which contains over a million known species of insects with distinct life history. They have been found in almost every possible nutritional niche on Earth. Therefore, the knowledge from *D*. *melanogaster* and mosquitoes cannot be simply generalized to the notorious agricultural pests. Given the pivotal effects of gut microbiota on hosts, it is of great significance to investigate the mechanisms underlying the interactions between pests and their gut microbiota which will uncover the novel potential target to boost the development of new promising management tactics on these notorious pests by disrupting these symbiotic associations.

Red palm weevil (RPW), *Rhynchophorus ferrugineus* Olivier, is the most devastating insect pest of palm trees worldwide (Giblin-Davis et al., 2013). This pest is native to South Eastern Asia, but it has spread to the Middle East, Africa and the Mediterranean due to the international exchange of infected plant materials. More recently, RPW has also been found in China, Japan, Australia, and the Caribbean (Zhang et al., 2003; Wang et al., 2015). Since its invasion in China, RPW has killed almost 20,000 coconut trees and its infestation area is over 10,000 km^2^, and thus has seriously threatened the ecological security of Chinese coastal system (Shi et al., 2014; Ge et al., 2016). RPW larvae are the major infestation agents which feed on the tender tissue inside the trunk which have abundant carbohydrates but poor assimilable nitrogen sources (Nagnan et al., 1992). Currently, it has been found that RPW gut harbors a complex bacterial community which can degrade polysaccharides and sucrose (Butera et al., 2012; Jia et al., 2013; Montagna et al., 2015). Furthermore, changes in the composition of gut microbiota can significantly influence the nutrition metabolism of this pest (Muhammad et al., 2017; Dawadi et al., 2018; Xiao et al., 2019). Because RPW gut microbiota is composed of a plethora of different bacterial species, distinguishing the impact of a specific bacterial species on host fitness is challenging. Interestingly, it is a powerful and effective way to study the intestinal mutualism by generating germ free (GF) and gnotobiotic experimental models in *D*. *melanogaster* and mice (Shin et al., 2011; Nicholson et al., 2012).

In the present study, we developed a protocol on how to generate GF RPW larvae firstly. And then the exact effects of gut microbiota on the development, body mass gain and survival rate of this pest were determined with this GF RPW model. Furthermore, the concentration of some important nutritional indices, containing hemolymph protein, glucose, triglyceride, and trehalose which can indicate the nutrition and metabolic status of animal host well (Ridley et al., 2012; Newell and Douglas, 2014), were measured to reveal the physiological consequences of gut microbiota on their host phenotype. It has been verified that *Enterobacter cloacae* can produce various glycolytic enzymes, containing cellulases, trehalases and other glucosidases, which participate in food digestion (Xia et al., 2017; Adhav et al., 2019). Moreover, *Lactococcus lactis* is a lactic acid bacterium which is involved in digesting and fermenting some plant polymers to improve insect nutrition acquisition (Passerini et al., 2013; Zhou H. et al., 2017). Both *E*. *cloacae* RPWL3 (access no. MF185375) and *L*. *lactis* RPWL8 (access no. MF185378) are present in RPW gut with higher abundance (Muhammad et al., 2017), so their influence on host nutrition status were determined by making gnotobiotic insects in this study. Collectively, we provided the evidence to show the promoting effects of gut microbiota on the development and body mass gain of RPW larvae by improving their nutrition metabolism. The generation of GF and gnotobiotic RPW larvae set the groundwork to facilitate the understanding of molecular physiological mechanisms that promote RPW microbial coexistence and the processes by which intestinal microbiota affect host fitness.

## Materials and Methods

### Insect Rearing and Maintaining

Lab RPW population were established and maintained from the adults which were trapped in Jinshan campus of Fujian Agriculture and Forestry university (119°30′ E, 26°08′ N) and Pingtan District (119°32′ E, 25°31′ N) of Fujian Province. The adults were fed with sugarcane stems in the incubator (Saifu ZRX-260, Ningbo Experimental Instrument Co., Ltd., China) at 27 ± 1°C, 75% relative humidity (RH) under the photoperiod of 12 h light/12 h dark, while larvae were reared under the following conditions: 27 ± 1°C, 75% RH and a photoperiod of 24 h dark. The fresh eggs, within 12 h after oviposition, were collected for dechorionation to generate GF insects. These collected eggs were divided into the following two groups: the dechorionated eggs which were processed to sequential washing by 10% sodium hypochlorite solution, 75% ethanol and sterilized water, others served as the control which was only treated with sterilized water. These treated eggs were moved to a sterilized Petri dish with autoclaved wet cottons for hatching and their hatching status were observed every 24 h. After dechorionation, the germfree individuals were always maintained in the laminar hood (Heal Force safe-1200LC) which can prevent the bacterial contamination from outside environment. The neonatal larvae were fed on sterile artificial diet (date palm tissue 8.0g, sucrose 8.0g, agar 6.0g, casein 8.0g, corn flour 10.0g, yeast extract 12.0g, avicel 5.0g, ascorbic acid as vitamin C 1.0g, potassium sorbate 0.4g, sodium p-hydroxybenzoate 0.2g, cholesterol 0.3g, choline chloride 0.25g, inositol 0.02g, and 220 ml distilled water) with or without antibiotic cocktail (Pu and Hou, 2016). Antibiotic cocktail (Kanamycin 150 mg/L, Tetracycline 150 mg/L, Gentamicin 150 mg/L, and Erythromycin 150 mg/L) and 1g ascorbic acid was added to the sterilized artificial diet when the temperature dropped to about 55°C. Each larva was provided with 3 g artificial food which was refreshed every 2 day. The axenic RPW individuals were generated as follows: the collected fresh eggs were washed in 10% sodium hypochlorite solution (NaClO) for 3–5 min, rinsed in 75% ethanol two times to remove the bacteria species on the egg surface, and then rinsed with sterilized water two times to exclude the potential effect of hypochlorite and ethanol on the following experiments. In the control group, the fresh eggs were only washed with sterilized distilled water by the same procedure. Subsequently, these treated eggs were transferred to the sterilized Petri dish (90 mm in diameter) containing autoclaved wet cottons for hatching. After dechorionation, the germfree individuals were always maintained in the laminar hood (Heal Force safe-1200LC) which can prevent the bacterial contamination from outside environment. All larvae were maintained at 27 ± 1°C, 75% relative humidity (RH) under the photoperiod of 24 h dark. However, the conventionally reared ones were maintained in the common incubator (Saifu ZRX-260, Ningbo Experimental Instrument Co., Ltd., China). Overall, there were four treatments: dechorionated eggs + food with antibiotics (GF), dechorionated eggs + food without antibiotics (DNA), non-dechorionated eggs + food with antibiotics (NDA), and non-dechorionated eggs + food without antibiotics (CR). Insects in the former three treatments were maintained in the laminar hood while the fourth group was reared in the incubator. To generate GF+B larvae through introducing the intestinal microbiota to GF larvae, 50 μl gut homogenates from CR larvae were poured on sterilized artificial food to feed them. Each treatment contained three replicates and three fifth instar larvae were used in a replicate.

The fifth instar GF larvae were employed to generate the gnotobiotic insects and they were transferred to sterilized food without antibiotics. *E*. *cloacae* RPWL3 and *L*. *lactis* RPWL8 were cultivated at 37°C overnight in nutrient broth (NB, tryptone 10 g/L, beef extract powder 5 g/L NaCl 10 g/L and pH 7.2). Then 50 μl bacterial suspensions (OD_600_ = 0.15) of two species were spread on sterilized food to feed the fifth instar GF RPW larvae. During the generation of gnotobiotic insects, their food was refreshed every 2 day. For the control groups, the same volume of NB was introduced on the food to feed insects. Seven day later, four insects were randomly selected from each group to verify the load of two introduced bacterial species. Gut homogenates of these insects were prepared, poured on nutrient agar (NA, tryptone 10.0g/L, beef extract powder 3.0g/L, NaCl 5.0g/L, 15.0g/L agar and pH 7.2) and incubated as above. Bacterial species was determined by 16S rRNA-based PCR diagnosis and the number of colony-forming units (CFUs) was counted 24 h after incubation.

### Verification of Germfree RPW Individuals With Culture-Dependent and–Independent Assays

To verify if there were some commensal bacteria in the gut of RPW larvae, three Fifth-instar larvae were randomly dissected to collect guts. Before dissection, each individual surface was cleaned with 75% ethanol, followed by three rinses in the sterilized water. The whole gut of each insect was pulled out in a clean Petri dish with sterilized forceps. Each gut was put in 1 ml sterile PBS as a replicate and homogenized by the Scientz-48 tissue lyser (Ningbo Scientz BioTech. Co. Ltd, China). Each treatment comprises at least three replicates. In the culture-dependent assays, 100 μl gut homogenate was poured on the nutrient agar media in triplicate after serial dilution (10^-1^ to 10^-4^) and incubated aerobically at 37°C for 24 h. Finally, the number of bacterial colony forming units (CFUs) was counted for further analysis.

By using a DNeasy Blood & Tissue Kit (Qiagen), total gut bacterial DNA was extracted from RPW larvae in the four treatments according to the procedures as described by Muhammad et al. (2017). To detect if there were any bacteria in RPW gut with a highly sensitive way, PCR diagnosis with bacterial 16S rRNA primers (27F: 5′-AGAGTTTGATCATGGC TCAG-3′, 1492R: 5′-TACGGYTACCTTGTTACGACTT-3′) was performed with total reaction volume of 25 μl reaction system, being composed of 50 ng template DNA, 1 μl forward primer, 1 μl reverse primer and 12.5 μl of 2X Taq PCR Mastermix (Beijing Tiangen BioTech Co., Ltd., China). PCR reactions were run with 1 cycle of denaturation at 94°C for 3 min, 30 cycles at 94°C for 30 s, amplification at 55°C for 30 s, and a dissociation step. Finally, the PCR products were determined by electrophoresis on 1% agarose gel stained with ethidium bromide and visualized under UV light.

### Effect of Gut Microbiota on RPW Physiological Traits and Nutrition Status

The instar and survival of each larva were observed daily and its body mass was also measured by an electric microbalance (Mettler Toledo AL104) to the accuracy of 0.01 μg. The seventh instar larvae were used to collect the hemolymph for the quantification of four nutritional indices, including protein, glucose, triglyceride (TAG) and trehalose concentration. Before hemolymph collection, each larva was washed with running water to remove excrement and food particles, and then anesthetized for 3–5 min on ice for immobilization. Subsequently, its epidermis was pierced by a fine sharp needle for collecting 50 μl of hemolymph per larva into a labeled 1.5 ml clean micro-centrifuge tube with 2 μl of 0.2% phenylthiourea (PTU) to inhibit the hemolymph coagulation. The protein content of each sample was analyzed with the BCA protein assay kit (Beijing Tiangen BioTech. Co., Ltd., China). Glucose was measured using Glucose Measurement Kit (Shanghai Rongsheng Biology Pharmaceutical Co., Ltd., China). TAG content was determined with Triglyceride assay kit (Zhejiang Dongou Diagnostic Products Co., Ltd., China). The concentration of trehalose was quantified with trehalose assay kit (Megazyme Bray, Co., Wicklow, Ireland), following the manufacturer’s instructions.

### Statistical Analysis

The differences in body mass, development duration and eggs hatching success between GF and conventionally reared (CR) group was detected by independent *t*-test. The variations of nutrition indices across different groups were assessed by analysis of variance (ANOVA) and multiple comparisons were conducted with Tukey’s HSD *post hoc* analysis. Before ANOVA or *t*-test, Kolmogorov-Smirnov test and test of homogeneity of variances was run for determining if our data has normal distribution and equal variances, respectively. If not, the data were transformed to meet the prerequisite of parametric tests. All analyses were performed using IBM SPSS Statistics (22.0). Log rank test procedure was used to determine effects of microbiota on survival test. The significance level to threshold was set at *P <* 0.05.

## Results

### The Generation and Verification of Germfree RPW Larvae

**FIGURE 1:**
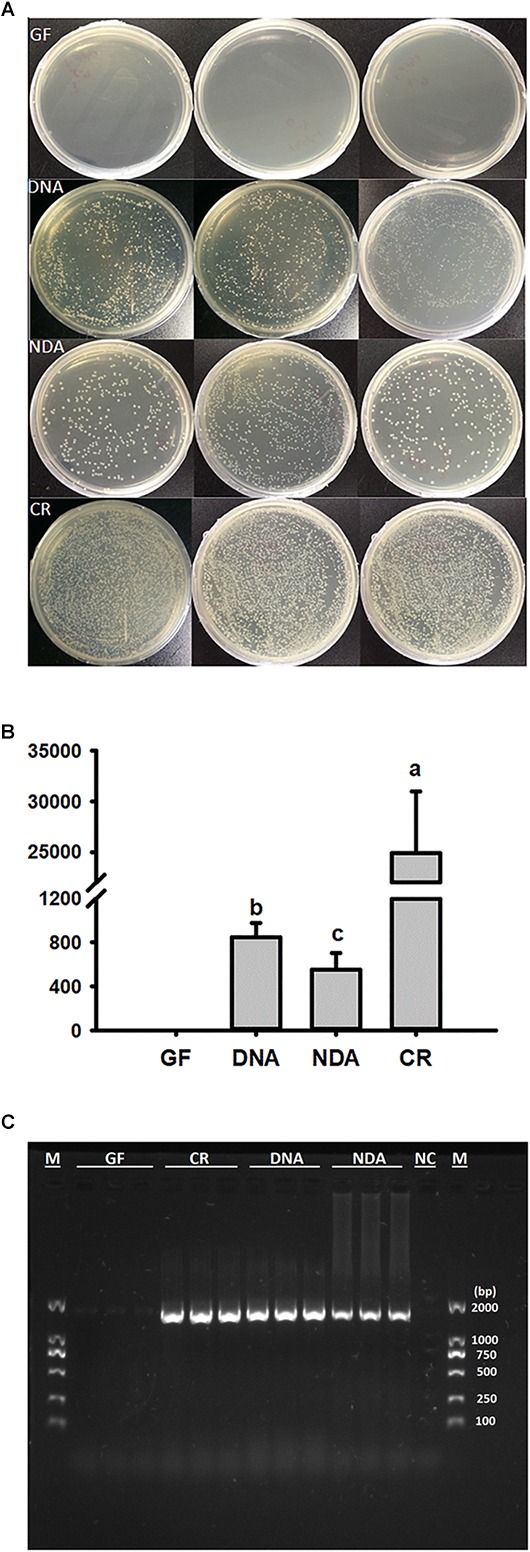
Verification of gut bacteria in the larvae of *Rhynchophorus ferrugineus* with culture-dependent **(A,B)** 10^-4^ dilution plating, and –independent methods **(C)**. GF, dechorionated eggs + food with antibiotics; DNA, dechorionated eggs + food without antibiotics; NDA, non-dechorionated eggs + food with antibiotics; CR, non-dechorionated eggs + food without antibiotics.

The culture-dependent validations revealed that the greatest quantity of bacterial colonies was found in guts of CR group, followed by DNA and NDA, while no bacterial colony was detected in GF group (Figure 1A,B). Moreover, PCR diagnosis based on bacterial 16S rRNA uncovered that the expected bands, with the size of about 1500 bp, only missed in the GF group, indicating that the guts of GF RPW larvae was not colonized by any bacteria species (Figure 1C). Therefore, RPW larvae without any intestinal bacteria species were successfully generated and maintained with our protocols.

### Effect of Gut Microbiota on Insect on Hatching Success, Survival and Body Mass

**FIGURE 2:**
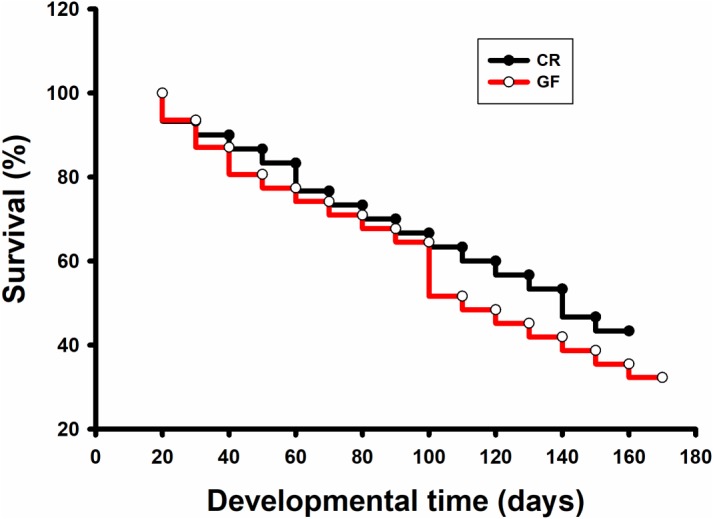
Survival analysis of conventionally reared (CR) and germfree (GF) *Rhynchophorus ferrugineus* larvae.

**FIGURE 3:**
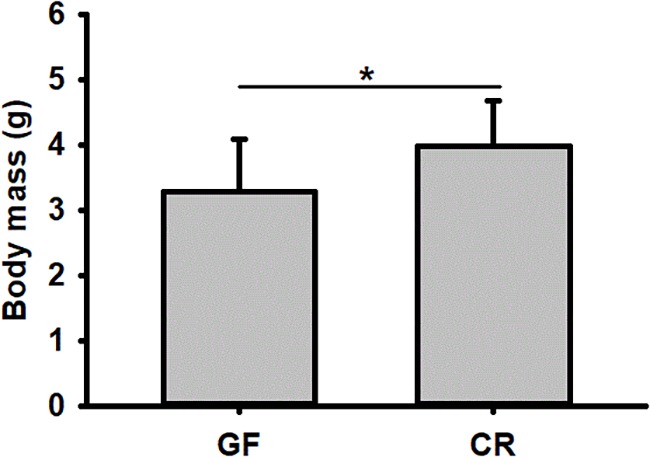
Body mass of conventionally reared (CR) and germfree (GF) *Rhynchophorus ferrugineus* larvae. ^∗^Indicates the significance between two groups.

No significant difference was detected in hatching success (*t*-test: *t* = 0.310, *P* = 0.91) and the time to hatching of dechorionated eggs and controls (2.59 ± 0.94 and 2.38 ± 0.61 day, *t*-test: *t* = -1.55, *df* = 26, *P* = 0.30, Table 1). The survival rate of RPW individuals to prepupa in the two groups were not significantly different from each other (log rank test *P* = 0.069; X^2^ = 3.2, Figure 2), indicating that the RPW-gut microbe symbiosis is facultative, not obligate. Furthermore, body mass of RPW GF larvae was significantly less than that of CR insects (*t*-test = -2.29, *df* = 22, *P* = 0.032). When compared to CR larvae, body mass of RPW GF individuals were decreased by 17.59% (Figure 3).

**Table 1:** Comparisons on the fitness parameters of conventionally reared (CR) and germfree (GF) larvae of *Rhynchophorus ferrugineus* Olivier.

Treatment	Hatching success (%)	Time to hatching (*d*)	Development time to prepupa (*d*)
CR	35.00	2.59 ± 0.17	159.92 ± 2.17
GF	38.75	2.38 ± 0.11	163.20 ± 4.23
	*P* = 0.256	*P* = 0.264	*P* = 0.005


### Effect of Gut Microbiota on the Concentration of Hemolymph Protein, Glucose, TG and Trehalose in RPW Larvae

**FIGURE 4:**
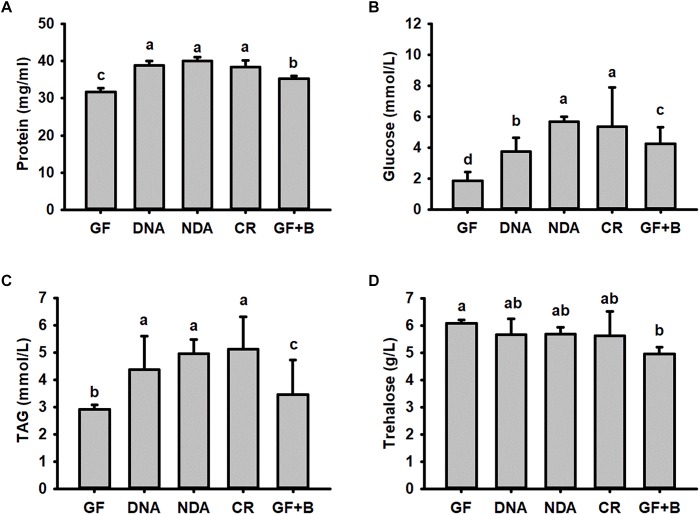
The content of hemolymph protein **(A)**, glucose **(B)**, triglyceride **(C)** and trehalose **(D)** in *Rhynchophorus ferrugineus* larvae with different treatments. GF, dechorionated eggs + food with antibiotics; DNA, dechorionated eggs + food without antibiotics; NDA, non-dechorionated eggs + food with antibiotics; CR, non-dechorionated eggs + food without antibiotics; GF+B, GF larvae established the interactions with gut bacteria by feeding gut homogenates of CR. The different letters above each graph indicate the significance across groups.

No significant differences were found in our assayed nutrient indices in RPW larvae from NDA and CR group (Figure 4), revealing that the introduction of antibiotic into artificial food had no significant effects on RPW nutrient indices. Interestingly, the concentration of hemolymph protein (ANOVA: *F*_2,20_ = 38.02, *P* < 0.001, Figure 4A), glucose (ANOVA: *F*_4,20_ = 615.31, *P* < 0.001, Figure 4B) and TAG (ANOVA: *F*_4,20_ = 527.84, *P* < 0.001, Figure 4C) in the CR individuals were significantly higher than those of GF individuals.

The concentrations of protein, glucose and TAG in GF+B larvae, with introduced gut microbiota, were still markedly lower than those of CR insects. But their contents were significantly increased in contrast with GF larvae (Figure 4). Therefore, these data confirmed that introducing intestinal bacteria into the guts of GF larvae could restore their nutritional status dramatically. Significant difference was also determined in the content of trehalose across different groups (ANOVA: *F*_4,20_ = 3.26, *P* = 0.033, Figure 4D). However, the content of trehalose of GF+B larvae was significantly lower than that of GF individuals. Taken together, these data suggested that intestinal commensal bacteria have profound effects on the nutrition metabolism of RPW larvae.

### Impact of *Lactococcus lactis* and *Enterobacter cloacae* Monoassciation With RPW Larvae on the Concentration of Hemolymph Protein, Glucose, TAG and Trehalose

**FIGURE 5:**
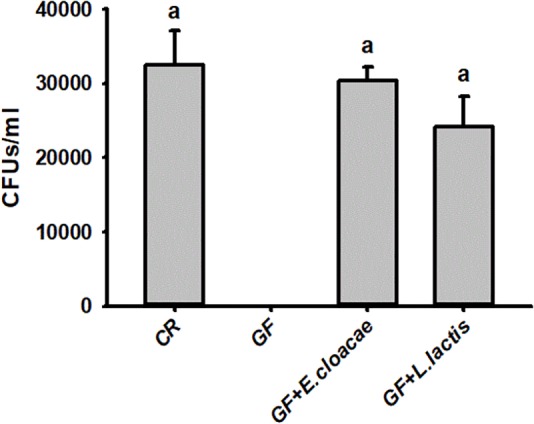
The CFU number of *Enterobacter cloacae* and *Lactococcus lactis* were harvested from the guts of *Rhynchophorus ferrugineus* gnotobiotic larvae.

**FIGURE 6:**
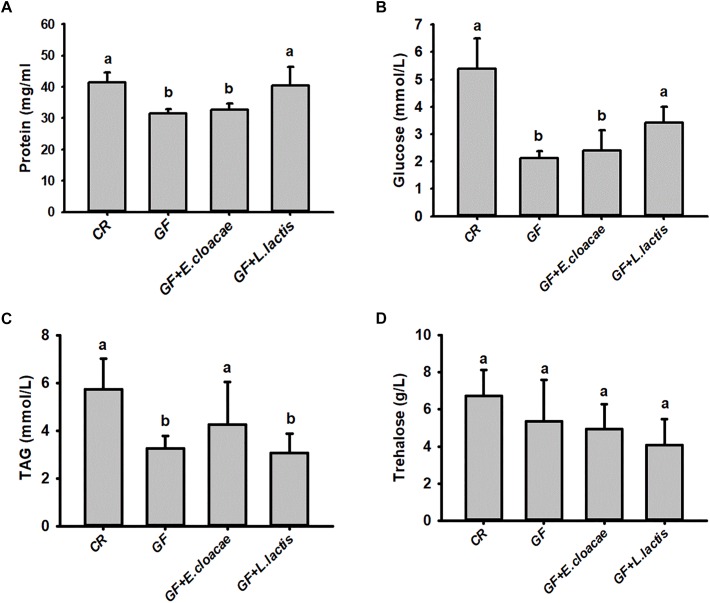
The level of hemolymph protein **(A)**, glucose **(B)**, triglyceride **(C)** and trehalose **(D)** in the gnotobiotic larvae inoculated with *Lactococcus lactis* and *Enterobacter cloacae*. The data on glucose **(B)** and triglyceride **(C)** were transformed by Log10 and Square root to meet the prerequisite of ANOVA, respectively. The different letters above each graph indicate the significance across groups.

The number of *E*. *cloacae* and *L*. *lactis*, being harvested from gut homogenate of gnotobiotic larvae was 30,317.66 ± 1913.98 CFUs/ml and 24150.00 ± 4084.42 CFUs/ml (Figure 5), respectively. These data indicated that gnotobiotic RPW larvae with *E*. *cloacae* and *L*. *lactis* were successfully established. Furthermore, we also found that gnotobiotic RPW larvae with *L*. *lactis* exhibited markedly higher protein content as compared to that of GF insects (ANOVA: *F*_3,12_ = 8.459, *P* = 0.003, Figure 6A), suggesting that *L*. *lactis* could restore the protein level to that of CR larvae. In contrast, the level of TAG (ANOVA: *F*_3,12_ = 12.357, *P* = 0.001, Figure 6B) and glucose (ANOVA: *F*_3,12_ = 13.215, *P* < 0.001, Figure 6C) in gnotobiotic larvae with *E*. *cloacae* were significantly improved in contrast to the GF individuals. But no significant difference was detected in trehalose content between gnotobiotic and GF groups (ANOVA: *F*_3,12_ = 1.985, *P* = 0.304, Figure 6D). Consequently, we provided the evidence to show that two gut bacteria species, *E*. *cloacae* and *L*. *lactis*, could play different roles in regulating the nutrition status of RPW larvae.

## Discussion

Recent evidence demonstrated that RPW harbor a complex gut microbiota with profound effects on food digestion and nutrition assimilation of this pest (Butera et al., 2012; Jia et al., 2013; Montagna et al., 2015; Muhammad et al., 2017). However, it is still unknown whether the specific bacterial community or the interactions between different bacterial groups mediate these physiological traits and the molecular dialogue that shapes host-microbiota mutualism because of the complexity of gut microbiota. To this end, GF RPW larvae were generated and maintained by our protocols reported here. Previous investigations have revealed that insect gut microbiota can be transferred vertically via depositing gut bacteria on the egg surface (Behar et al., 2008). Embryo dechorionation is an important step as it has been confirmed that eggshell layer carries microbes from adults (Sabat et al., 2015; Kolye et al., 2016; Kietz et al., 2018). Recently, it has been verified that *D*. *melanogaster* must consume bacteria from its food to establish and maintain its microbiome (Blum et al., 2013). In the present study, we provide the evidence to show that dechorionation and sterilized food with antibiotics are used together to successfully make RPW GF larvae. Dechorionation can remove the bacteria on egg surface, while antibiotics are able to prevent the proliferation of gut bacteria in the sterilized food. The generation of GF RPW larvae provides a tool for dissecting the different aspects of host-microbe interplay. More importantly, it has also laid a strong foundation for determining the precise role of specific bacteria on host by generating gnotobiotic model organism.

We found body mass of RPW GF larvae was less than that of CR insects and the development rate from eggs to GF prepupae was prolonged significantly as well, suggesting the promoting effect of gut microbiota on the growth and development of this pest. Additionally, our data uncovered that the content of hemolymph protein, glucose and triglyceride of GF larvae were significantly lower than that of CR insects. Candidate bacterial products are acetic acid and lactic acid, which are derived from *Acetobacter* and *Lactobacillus* species, and are known to reduce the digestibility of starch and other carbohydrates by mammals (Johnston et al., 2010; Matzkin et al., 2011). *L*. *plantarum* in *D. melanogaster* can enhance its dietary protein digestion and amino acid intake by upregulating the expression of intestinal peptidases (Erkosar et al., 2015). Therefore, introducing gut bacteria into RPW GF larvae could restore the above nutrition indices markedly. In this way, these data suggest that less body mass gain of GF larvae is the resulting effect of gut microbiota absence-mediated nutrition metabolic defects. This is consistent with the recent reports on the impact of gut microbiota on *D*. *melanogaster* (Shin et al., 2011; Storelli et al., 2011, 2018) and honeybee *A*. *mellifera* (Zheng et al., 2017). To note, previous investigations have also revealed that gut microbiota can enhance body mass gain and development via modulating insulin signaling (Shin et al., 2011; Storelli and Leulier, 2016; Zheng et al., 2017). Unfortunately, what gut microbiota-derived chemical signals result in the promoting effects on insect host phenotype remains largely unknown. In mammals, it has been verified that gut microbiota-derived short-chain fatty acids, such as acetate, butyrate and propionate, serve as the key metabolites to contribute to host health and disease via providing energy and modifying chromatin status (Donohoe et al., 2011; Lee and Hase, 2014; Koh et al., 2016; Krautkramer et al., 2016; Fellows et al., 2018). More recently, Zheng et al. (2017) found that the honeybee gut bacteria produce short-chain fatty acids, being dominated by acetate and propionate, affects bee growth, gut physicochemical conditions and hormonal signaling. Yet the profile of gut microbiota-derived short-chain fatty acids and molecular mechanisms behind these short-chain fatty acids on the physiological traits of insects need further extensive elucidation.

In this study, we uncovered that feeding *L*. *lactis* to RPW GF larvae could elevate the protein content significantly. *L*. *lactis*, being involved in the production of fermented dairy products containing lactic acid (Bolotin et al., 2001), is present as a dominant species in RPW gut in July (Jia et al., 2013; Tagliavia et al., 2014). *Lactobacillus* has been used as model lactic acid bacteria because they are widely recognized as the potential health beneficial microbes in human gastrointestinal tract (Perpetuini et al., 2013; Drissi et al., 2014). More interestingly, *L*. *plantarum* can promote the growth of *Drosophila* under chronic undernutrition via enhancing the expression of intestinal peptidases and then increasing host dietary protein digestion and amino acid intake (Erkosar et al., 2015). Furthermore, *L*. *plantarum*-derived D-alanylated teichoic acids can ensure the optimal intestinal peptidase expression (Matos et al., 2017). However, the mechanisms underlying the promoting effects of *L*. *lactis* on the nitrogen nutrition assimilation of this pest remain elusive. Because *L*. *lactis* is a lactic acid bacterium as well, we postulate that this commensal bacterium possibly enhance host physiology via the similar actions.

*Enterobacteriaceae* has been determined as the dominant populations in the RPW gut (Jia et al., 2013; Tagliavia et al., 2014; Muhammad et al., 2017). Members of *Enterobacteriaceae* are known as nitrogen fixers (Behar et al., 2005; Morales-Jiménez et al., 2009; Ben-Yosef et al., 2014). Here, we also found that RPW gnotobiotic larvae with *E*. *cloacae* exhibited the dramatically recapitulated level of hemolymph glucose and triglyceride, being consistent with the observed effects of *E*. *cloacae* B29 in gnotobiotic mice (Fei and Zhao, 2013). *E*. *cloacae*, producing various carbohydrate modifying and glycolytic enzymes like cellulases, trehalases and other glucosidase to facilitate insect nutrition acquisition, was found to be the most abundant species of gut bacteria in *P*. *xylostella* larva (Xia et al., 2017). Recently, it has been unveiled that *E*. *cloacae* can assist in trehalose hydrolysis by secreting trehalase to modulate energy metabolism in *P. xylostella* (Adhav et al., 2019). In this context, it can be deduced that *E*. *cloacae* might release some carbohydrate hydrolytic enzymes into RPW larva gut to facilitate the digestion of food polymers to regulate its energy metabolism. Collectively, the mechanisms behind how *E*. *cloacae* and *L*. *lactis* affect nutrition metabolism of RPW need be defined further with these gnotobiotic models.

## Conclusion

In summary, we determined that RPW has established facultative mutualistic interplays with its gut microbiota. Gut bacteria promote the development and growth of RPW via modulating nutrition metabolism of this pest. Additionally, the monoassociation of two dominant gut bacteria, *L*. *lactis* and *E*. *cloacae*, could restore the content of some nutrition in contrast to GF RPW larvae. Given the importance of gut microbiota on RPW physiology, it is a very promising alternative way to control this pest by disrupting the interactions between RPW and its gut microbiota.

## Ethics Statement

*Rhynchophorus ferrugineus* is exempted from above mentioned requirements.

## Author Contributions

ZS conceived and designed this project. PH, AM, TJ, RX, and XY performed the experiments. ZS, PH, AM and YH performed the analysis and wrote this manuscript. All authors have approved the final version of the manuscript.

## Conflict of Interest Statement

The authors declare that the research was conducted in the absence of any commercial or financial relationships that could be construed as a potential conflict of interest.
